# Efficacy of Lidocaine Infusion in High-Risk Vascular Surgery—A Randomized, Double-Blind, Placebo-Controlled Single-Center Clinical Trial

**DOI:** 10.3390/jcm12062312

**Published:** 2023-03-16

**Authors:** Dariusz Gajniak, Konrad Mendrala, Tomasz Cyzowski, Michał Polak, Danuta Gierek, Łukasz J. Krzych

**Affiliations:** 1 Department of Anaesthesiology and Intensive Care, Upper Silesian Medical Centre, Medical University of Silesia, 40-752 Katowice, Poland; 2 Department of Anaesthesiology and Intensive Care, Faculty of Medical Sciences in Katowice, Medical University of Silesia, 40-752 Katowice, Poland

**Keywords:** analgesia, aorta, high-risk surgery, lidocaine, opioid-sparing anesthesia, pain management, perioperative, vascular surgery

## Abstract

Background: In perioperative pain control, adjuvants such as lidocaine can reduce opioid consumption in a specific type of surgery. The aim of this single-center prospective double-blinded randomized controlled trial was to determine opioid consumption in the perioperative period in patients receiving continuous lidocaine infusion. Methods: Patients undergoing elective abdominal aorta and/or iliac arteries open surgery were randomized into two groups to receive 1% lidocaine or placebo at the same infusion rate based on ideal body weight (bolus of 0.15 mL/kg during the induction of anesthesia followed by continuous infusion of 0.2 mL/kg/h during surgery; postoperatively 0.1 mL/kg/h for 24 h) additionally to standard opioid analgesia. Results: Total opioid consumption within 24 h after surgery was 89.2 mg (95%CI 80.9–97.4) in the lidocaine and 113.1 mg (95%CI 102.5–123.6) in the placebo group (*p* = 0.0007). Similar findings were observed in opioid consumption intraoperatively (26.7 mg (95%CI 22.2–31.3) vs. 35.1 mg (95%CI 29.1–41.2), respectively, *p* = 0.029) and six hours postoperatively (47.5 mg (IQR 37.5–59.5) vs. 60 mg (IQR 44–83), respectively, *p* = 0.01). Conclusions: In high-risk vascular surgery, lidocaine infusion as an adjunct to standard perioperative analgesia is effective. It may decrease opioid consumption by more than 20% during the first 24 h after surgery, with no serious adverse effects noted during the study period.

## 1. Introduction

Open aortic surgery is an example of a procedure associated with extensive tissue trauma, high intensity of postoperative pain, and high-risk of cardiovascular complications [1]. According to the ESC/ESA (European Society of Cardiology/European Society of Anaesthesiology), cardiac risk in these procedures is considered very high and exceeds 5% [2,3]. Frailty of the patients, combined with significant fluid shifts, bleeding, hemodynamic instability, and an aggravated stress response, may increase the risk of severe cardiological complications even more. In addition, severe postoperative pain diminishes patient satisfaction, restricts the possibility of early rehabilitation, and increases the frequency of adverse events (mostly cardiovascular and pulmonary complications), affecting postoperative morbidity and mortality. Proper postoperative pain control is one of the key pillars of postoperative care, therefore, optimizing strategies and an individualized approach to patient’s needs are of paramount importance.

Guidelines of various anesthesiology societies regarding pain management differ, but unanimously emphasize the role of multimodal analgesia [4,5,6]. This method involves using various drugs with different mechanisms of action, both peripheral and central, which allows more effective treatment and fewer adverse events compared to a single drug method. This concept has been repeatedly investigated in clinical trials. One of its main advantages is the ability to significantly reduce the use of opioids, both in the intraoperative period and in postoperative care [7]. 

The diversity of surgical procedures, the broad range of regional anesthesia techniques and non-opioid adjuvants are the main issues challenging multimodal analgesia. Intravenous infusion of lidocaine (LI) is one of the recommended components of a multidirectional strategy. It may reduce the amount of opioids used and thus reduce the frequency of their side effects including short and long-term consequences. There is still no conclusive data in the literature assessing the utility of lidocaine in major vascular surgery [8]. We hypothesize that in major vascular surgery performed transabdominally, perioperative opioid consumption can be reduced with LI. The aim of our study was to investigate the efficacy of the lidocaine infusion as an adjuvant to the standard opioid-based analgesia in patients undergoing high-risk vascular surgery. 

## 2. Materials and Methods

The study was a single-center, double-blinded, randomized controlled clinical trial. The study was approved by the Ethics Committee of the Medical University of Silesia in Katowice, Poland (KNW/0022/KB1/1/19), and was registered in the ClinicalTrials.gov online database (NCT04691726). The study was conducted from February 2019 to July 2022. There were no violations of the study protocol after trial commencement. A CONSORT statement was applied for appropriate reporting.

### 2.1. Participants

All adult patients were recruited from the Vascular Surgery Unit in the Upper Silesian Medical Center in Katowice, Poland. After written informed consent was obtained, participants scheduled for open vascular surgery on the abdominal aorta and/or iliac arteries were consecutively screened and enrolled. The surgery was performed transperitoneally through a midline incision from the xiphoid process to the pubic symphysis. 

**Figure 1:**
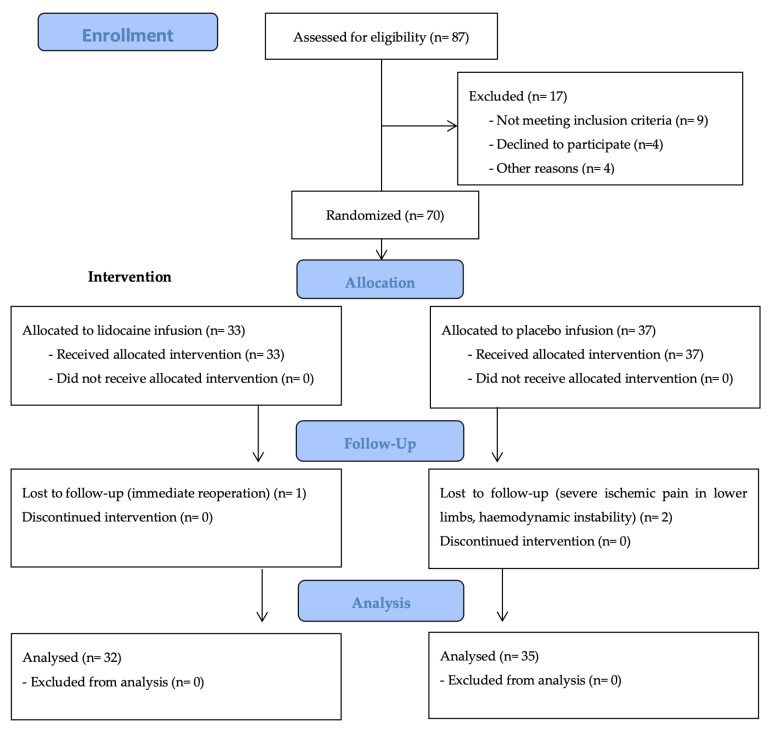
CONSORT chart.

Exclusion criteria were: lack of consent (or inability to receive it), urgent or emergency cases, contraindications for administration of lidocaine (according to Summary Product Characteristics) [9], preoperative administration of any pain relief medication, second or third-degree heart block, implantable cardiac stimulator, chronic atrial fibrillation, antiarrhythmic medication on a regular basis (excluding b-blockers), heart failure with reduced ejection fraction (HFrEF < 30%), epilepsy or any episode of seizure in the past, chronic renal failure (CKD 3–5), chronic liver failure (Child-Pugh class B or C), myasthenia gravis, hypoproteinemia, cognitive or mental dysfunction hindering cooperation. The patient flow diagram is shown in Figure 1. 

### 2.2. Pharmacological Intervention Design

All enrolled patients were randomized into two groups—group A (intervention, perioperative continuous infusion of 1% lidocaine) and group B (control, perioperative continuous infusion of placebo). The lidocaine/placebo regimen was based on Polish recommendations on perioperative pain treatment—the initial preoperative lidocaine bolus of 1.5 mg/kg, followed by continuous infusion of 1–3 mg/kg/h intraoperatively, and the dose of 1–3 mg/kg/h maintained for 24–48 h after surgery [6]. The unblinded coordinator of the randomization process (the person not involved in the participant’s perioperative care) was responsible for preparing a set containing, respectively, 40 mL of 1% lidocaine or 40 mL of 0.9% NaCl (placebo) before and after surgery. The syringes were coded with individual numbers, all labeled “Test Drug” and provided to the anesthetic team involved in the participant’s treatment and care. 

Dosing was based on the patient’s ideal body weight (IBW) and performed according to the following protocol: loading dose before induction of general anesthesia with an intravenous bolus of the “test drug” 0.15 mL/kg IBW given over 5 min, followed by continuous intravenous infusion of the “test drug” 0.2 mL/kg IBW/h intraoperatively. Postoperatively, the infusion was continued with a ratio 0.1 mL/kg IBW/h for 24 h. 

### 2.3. Randomization

All patients scheduled for surgery were assessed by a trial investigator (anesthesiologist). After receiving a detailed explanation of the study protocol and providing written informed consent, the participants were reported to the study coordinator. A simple randomization was performed using a computer-generated random number list, according to which the participants were assigned into group A or B (www.random.org). All data regarding the patients’ assignment to the respective groups was available only to the coordinator of the study. 

### 2.4. Patient Monitoring and Data Collection

Intraoperatively, the following parameters were monitored with a vital signs monitor Datex-Ohmeda F-CM1-05 (GE Healthcare, Helsinki, Finland) and recorded in a real-time manner using dedicated software (Vital Signs Capture Wave v1.006; 2018 John George K.) connected to the computer: invasive blood pressure (IBP), anesthetic agent concentration monitoring (MAC, minimal alveolar concentration), pulse oximetry (SpO2), capnometry (etCO2) and depth of anesthesia monitoring (state entropy (SE) and response entropy (RE)). In addition, patients’ comorbidities, American Society of Anesthesiology-Physical Status (ASA-PS), cardiac and respiratory risk according to Vascular Quality Initiative calculators (VQI), time, and the type of surgery were recorded.

### 2.5. General Anesthesia

All surgical procedures were performed under general anesthesia (GA) by an anesthetist experienced in vascular surgery. All patients received premedication with midazolam orally (Dormicum, Roche SA, Beerse, Belgium). We used doses ranging between 0.05–0.2 mg/kg, modified individually according to the clinical assessment of the patient’s frailty. 

A superficial forearm vein was cannulated with a peripheral catheter (Introcan Safety, Braun Medical Inc., PA, USA). For preemptive analgesia, all participants received 1 g of paracetamol i.v. (Paracetamol Kabi, Fresenius Kabi, Ostrava, Czech Republic) before the induction of general anesthesia. Patients scoring above 2 points in the Apfel score [10] received ondansetron 8 mg i.v. (Ondansetron, Bluefish Pharmaceuticals AB, Stockholm, Sweden). Induction of GA was performed by intravenous administration of 10–20 mcg/kg fentanyl citrate (Fentanyl WZF, Polfa Warszawa, Poland) and 1–2 mg/kg Propofol (Provive, Baxter Holding B.V., Utrecht, Holland); while 0.5 mg/kg atracurium (Tracrium, GlaxoSmithKline plc, Brentford, United Kingdom) was used for endotracheal intubation (7.5–9.0 mm endotracheal tube, Zarys, Zabrze, Poland). Anesthesia was maintained with Dräger Primus (Dräger, Lubeck, Germany) and inhaled desflurane (Suprane, Baxter International Inc., IL, USA). The depth of anesthesia was monitored with state entropy (GE Healthcare Helsinki, Finland) and maintained between 40–60. A muscular blockade was achieved with a repeated dose of atracurium. Boluses of fentanyl (FNT) were administered if needed, as described below. Mechanical ventilation was adjusted to an end-tidal carbon dioxide pressure (etCO_2_) between 35–45 and a SpO_2_ between 95–100%. The tidal volume was 6–8 mL/kg, respiratory rate 10–18 breaths/min, positive end-expiratory pressure (PEEP) 5 cm H_2_O, an inspired oxygen fraction ranging from 0.5–1.0 (FiO_2_). Cannulation of the radial or brachial artery (BD Arterial 20 G, BD Medical, Franklin Lakes, NJ, United States) was performed after the induction of anesthesia. Based on the anesthesiologist’s preferences the central venous cannulation (Arrow, Teleflex, Wayne, PA, USA) was placed under ultrasound guidance into the right or left internal jugular vein.

Intraoperative nociceptive response management was based on the values of the vital signs. Systolic blood pressure (SBP) within the range from 100 to 160 mmHg and mean arterial pressure (MAP) > 70 mmHg was regarded as a target value (TAV). In case of a sudden increase in BP or HR over TAV associated with painful stimulation from the operating field, the following actions were initiated:

- SBP or MAP below the TAV limit: fluid challenge iv with balanced crystalloids was commenced. If the patient was not responding to the fluid challenge, ephedrine in titrated doses of 5–10 mg was given intravenously to the maximal dose of 25 mg. If still ineffective, continuous infusion of noradrenaline was commenced.
- SBP above the limit: if painful response present, an i.v. bolus of FNT in titrated doses was given to a maximal dose of 200 mcg to achieve an SBP decrease to the TAV. If ineffective, the re-assessment was performed: painful stimulation present—FNT i.v.; no painful stimulation—urapidil in titrated doses of 5 mg i.v. administered to the effect (to maintain SBP within TAV).
- If the SBP or MAP was above the limit associated with the clamping of the aorta, urapidil was given in titrated doses as above.

The number of interventions, SBP values, intraoperative fluid therapy (total amount and type of fluids, blood products), and the total dose of vasoactive agents were recorded. 

### 2.6. Postoperative Care

After emerging from anesthesia, each patient received a titrated dose of morphine i.v. with a target score of <4 points on the visual analog scale (VAS), followed by a continuous infusion at a rate adjusted to the initial bolus. Additionally, each patient received metamizole 2.5 g i.v. twice daily (Pyralgin, Polpharma, Warsaw, Poland). After the surgery, patients were transferred to the post-surgery high-dependency unit (HDU). Pain management in the postoperative period was based on the Nurse Control Analgesia (NCA) method. Infusion of the “test drug” was stopped 24 h after the surgery.

The efficiency of analgesic treatment was assessed 4-hourly, with the target value < 4 points (assessment performed by HDU vascular surgery nurses). In case of pain exceeding 4 points on the VAS scale, the patient received an additional bolus of 3 mg of morphine i.v. A reassessment was performed after 15 min. If no improvement was achieved, a second bolus of 3 mg of morphine i.v. was administered followed by an increase in the morphine infusion rate by 1 mg/h. 

### 2.7. Sample Size Determination

Sample size was calculated from the equation for two independent groups of equal size (as per P. Armitage and G. Berry) [11]. We assumed a clinically significant difference in 24 h morphine consumption for 30 mg. Standard deviations for opioid consumption were calculated based on literature data and our experience [12]. We assumed alpha 0.05 and beta 80%. Thirty-one patients per group was calculated as the minimum sample size. 

### 2.8. Outcome Measures

The primary outcome was opioid consumption during the first 24 h. We assessed the opioid administration during general anesthesia and total postoperative opioid consumption within the first 6 and 24 h (including the period of GA). Doses of all opioids used were converted into the equivalent dose of morphine as per the formula 0.1 mg of fentanyl i.v. is equal to 10 mg of morphine i.v. [13]. Additionally, the anesthetic agent consumption during GA was analyzed. The secondary outcome was the occurrence of adverse medical events: cardiovascular complications (myocardial infarct/non-fatal acute coronary syndrome, cardiac arrest), pulmonary complications (pneumonia, respiratory insufficiency requiring mechanical ventilation), severe deterioration in the patient’s general condition resulting in transfer to ICU, all-cause mortality and an incidence of postoperative delirium (POD). We performed a telephone follow-up of the discharged patients. The total observation time was 30 days. Occurrence of POD was assessed with the Confusion Assessment Method in Intensive Care Unit (CAM-ICU) for the first 24 h after surgery. 

Any side effects in the postoperative period potentially associated with lidocaine administration (according to Summary Product Characteristics) were observed and recorded during the first 24 h after surgery. Length of hospitalization (LOH) was defined as the period from the day of surgery to the discharge home. Patients transferred to other units due to a medical condition were excluded from the analysis.

### 2.9. Statistical Analysis

For statistical analysis, we used StatsDirect 3.1 software (StatsDirect LDT, Birkenhead, United Kingdom). The distribution of variables was assessed with the Shapiro–Wilk test and QQ plots. Quantitative variables were presented as the mean and standard deviation (SD) or median and interquartile range (IQR, interquartile range). Qualitative variables were presented as absolute values and percentages. Differences between groups were assessed using the Student’s *t*-test or Mann–Whitney U test. For qualitative variables, contingency tables and the chi-square or Fisher’s exact test were used. We assumed *p* < 0.05 to be statistically significant.

## 3. Results

Sixty-seven patients undergoing major vascular surgery were randomized. Thirty-two patients were assigned to receive lidocaine infusion and thirty-five to receive placebo. All patients received their assigned treatment according to the protocol. One participant in the intervention group was lost to the follow-up due to urgent reoperation. Two patients from the placebo group were excluded from the analysis due to extreme intraoperative hemodynamic instability and severe ischemic pain in lower limbs. 

Detailed patients’ characteristics are shown in Table 1. Participants in both groups were similar except for the presence of diabetes mellitus type 2.

**Figure 2:**
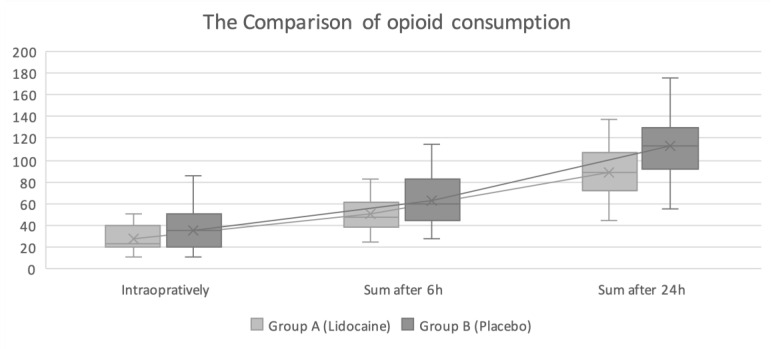
Comparison of opioid consumption between the groups at three time points. Range, medians and IQR or mean and 95%CI are marked on the graph.

Intraoperative opioid consumption in the lidocaine group was significantly lower than in the placebo group—26.7 mg (95%CI 22.2–31.3) vs. 35.1 mg (95%CI 29.1–41.2), respectively, (*p* = 0.029). The same result was observed regarding total opioid consumption within the first 6 h for lidocaine and the placebo group at 47.5 mg (IQR 37.5–59.5) and 60 mg (IQR 44–83), respectively, (*p* = 0.01), and 24 h after surgery at 89.2 mg (95%CI 80.9–97.4) and 113.1 mg (95%CI 102.5–123.6), respectively, (*p* = 0.0007). The results are shown in Figure 2.

Volatile anesthetic consumption was significantly lower in the lidocaine group in comparison to the placebo group—the median MAC of desflurane was 0.8 (IQR 0.7–0.9) vs. 0.9 (IQR 0.8–1.0), respectively, (*p* = 0.003). There were no differences in the depth of anesthesia between the groups. The median of the operating time was equal in both groups (175 min, *p* = 0.55). The characteristics of intraoperative interventions and the type of surgical procedures are shown in Table 2. Cross-clamping was sub-renal in all procedures that required it.

Postoperative delirium (POD) was diagnosed in one (3%) patient in the lidocaine group vs. six (17%) patients receiving placebo, which was not statistically significant (*p* = 0.1). 

Considering the median postoperative length of stay in the lidocaine and placebo group was six (IQR 5–8) and seven (IQR 6–10) days, respectively (*p* = 0.1). Two patients from the lidocaine group were removed from this sub-analysis (one transferred to ICU and one to the neurology department) and four patients from the placebo group, due to urgent transfer to other medical units (one to ICU, two to cardiology and one to the neurology department). 

There was no need to stop the infusion of lidocaine during the study time. Only a few minor lidocaine side effects were observed, as shown in Table 3. No auditory changes, tinnitus, perioral numbness, metallic taste, bradycardia or arrhythmia were observed. Three patients in group A and one in group B received ondansetron intraoperatively (*p* = 0.34). Medical major events during 30-days observation are shown in Table 4. 

## 4. Discussion

To our best knowledge, this study is the first randomized-controlled trial confirming the effectiveness of lidocaine infusion in high-risk vascular surgery. In the extensive surgery, including major vascular procedures, the standard approach to perioperative pain control is opioid administration. As for opioids, the price paid for the high effectiveness in pain management is the risk of classic side effects, such as excessive sedation, respiratory disorders, gastrointestinal motility disorders, nausea, and vomiting [14]. As a result, finding the right balance between adequate pain control and a low risk of side effects may be difficult. The trend toward decreasing opioid administration leads to a multimodal approach, including lidocaine infusion. 

The reduction in analgesic requirements during lidocaine infusion seen in our study is consistent with current literature [8,12,15,16,17]. However, all of the published studies focused on abdominal surgery without vascular involvement, and most of them did not provide detailed information on the patients’ comorbidities and ASA-PS scale score. Therefore, there are no publications we could compare our results directly with, in terms of the type of surgery and the patients’ population.

One of the most important trials on the use of lidocaine infusion for major abdominal procedures is the RCT by Kopper et al., in which the authors showed a reduction in opioid use during the 72 h postoperative period (159.0 mg ± 73.3 for placebo vs. 103.1 mg ± 72.0 for lidocaine) [12]. Our study also demonstrated a more than 20% reduction in opioid consumption in the first 24 h after surgery.

In contrast, a comprehensive meta-analysis by Weibel et al. questioned the clinical relevance of lidocaine infusion as an opioid sparing strategy, both during the early postoperative period (1–4 h) and 24–48 h after surgery [8]. The authors pointed out the low quality of evidence due to inconsistency and imprecision, which made it impossible to formulate a final recommendation. First of all, the analyzed data included a variety of surgical procedures and only 22 of 68 studies concerned open abdominal surgery. A sub-analysis of this population showed that lidocaine infusion reduced postoperative opioid consumption but the clinical significance of this finding remains unclear. One possible reason for the discrepancy is that different studies used different protocols for infusing lidocaine, with doses ranging from 1 mg/kg/h to 5 mg/kg/h, and infusions beginning and ending at different time points. The dosing strategy used in our project was in line with the recommendations included in the consensus of the Regional Anaesthesia Section of the Polish Society of Anaesthesiology and Intensive Therapy, as described in the methodology [6].

As an alternative to nonopioid adjuvants in pain management, wound infiltration with local anesthetics, peripheral nerve blocks, fascial blocks and central blocks (mainly epidural anesthesia) can be used [18]. Continuous epidural anesthesia may provide excellent results in pain management and, therefore, is widely used in fast-track protocols [19], but its implementation is not without potential complications. In vascular surgery, high-dose heparin administration brings the risk of hematoma formation at the site of catheter implantation, which may have catastrophic consequences resulting from spinal cord compression, also being a potential source of infection [20]. Although the epidural anesthesia technique is still preferred in some centers, it is not the method used in our department. 

Among other intravenous adjuvants, ketamine is one of the most potent, providing analgesia via the opioid receptors and acting as a co-analgetic. We resigned from ketamine administration because of its effect on EEG signal interpretation, while our study assumed maintaining a similar level of anesthesia between the groups assessed by entropy [21]. Magnesium infusion may also be an option for patients undergoing vascular surgery, although despite the significant reduction in opioid consumption the possibility of hypotonia must be considered [22].

In our study, significant reductions in desflurane doses were observed in the lidocaine group, with a comparable depth of anesthesia between both groups. These observations are consistent with existing data [15,16,17,23,24], although in some of the studies, a different anesthetic agent was used (sevoflurane) [16,23,24] and the depth of anesthesia was monitored using the BIS index, not entropy [15,16,23]. Desflurane was used in only two available studies [15,25]. Kuo et al. showed a significant reduction in volatile anesthetic use, although a different dosing regimen of lidocaine was applied; anesthetic concentrations were presented as end-expiratory concentration values, not age-related MAC, and the depth of anesthesia was monitored using auditory evoked potentials [25]. In the study of Lauwick et al., the reduction in desflurane consumption was also significant, but a different method of EEG monitoring was adopted, and the depth of anesthesia was adjusted according to the intraoperative MAP values and heart rate [15]. 

Although lidocaine’s opioid-sparing effect has been well-documented, its mechanism of analgesic action remains unclear. Lidocaine has the ability to block Na+ and K+ ion channels as well as regulate both intracellular and extracellular calcium concentrations. By blocking ion channels in the dorsal root ganglion (DRG), lidocaine may inhibit the transmission of nociceptive information to the central nervous system (CNS). Lidocaine also acts on ligand-gated channels, modulating glycine receptors and inhibiting glutamate release from presynaptic endings of cortical neurons and the spinal cord. These mechanisms modulate neuronal membrane potential, affecting the frequency of discharge and the rate of conduction of action potentials in nerve fibers [26]. Apart from opioid-sparing properties, lidocaine also has an anti-inflammatory, antithrombotic effect and reduces the occurrence of primary and secondary hyperalgesia, which may be of great importance in vascular surgery [27]. Although major vascular surgery is associated with all of the above-mentioned properties, our study was not designed to analyze them. 

In terms of safety, no major and only a few minor side effects were observed. Drowsiness was the most frequent and was almost equally distributed between the groups. Interestingly, we observed a non-significantly lower incidence of POD in the lidocaine group diagnosed with the CAM ICU. Several diagnostic instruments for delirium diagnosis have been developed over the years based on the diagnostic criteria for delirium, such as the Delirium Symptom Interview, Saskatoon Delirium Checklist, Delirium Rating Scale—revised version (DRS-R-98), MDAS, CAM, and CAM-ICU. In our publication, we used the CAM-ICU because our personnel are trained to use it. It has low inter-observer variability and a short patient assessment time (about 1–2 min). Compared to the diagnosis of delirium made by experts based on DSM-IV criteria, CAM-ICU has a sensitivity of 95% to 100%, and a specificity of 93% to 98% [28]. We did not notice any major complications associated with the pain treatment strategy or symptoms of local anesthetic toxicity. What should be underlined is that the population enrolled to our study was additionally exposed to potential complications due to multimorbidity and chronic oral medication. Further, the length of hospitalization was shorter in the LI group which is consistent with the available literature [7,17]. 

## 5. Study Limitations

As initially registered on ClinicalTrials.gov, the study’s original design was to analyze the effect of lidocaine on both opioid/anesthetic consumption and hemodynamic stability. Due to COVID-19, the study population was limited and the focus was solely on opioid and anesthetic consumption. Therefore, hemodynamic parameters such as pulse pressure variations and stroke volume variations were not analyzed. 

The second limitation was a lidocaine regimen, which is still under debate at the time of writing. The design of our protocol was based on Polish National Recommendations, not international consensus. Lidocaine infusion in our study was adjusted to the ideal body weight, which is recommended by the International Consensus 2020 [29]. Previously conducted studies were based mainly on actual body weight, which is probably the source of additional misjudgment. 

Thirdly, the significant disproportion in the consumption of morphine obtained in the study, in favor of the intervention group, may result from the design of the methodology/procedure, where, instead of a PCA (patient-controlled analgesia) pump, an automatic syringe operated by staff and a base opioid infusion were applied. Patients, after major vascular surgery, are often weak and may have cognitive impairment, especially within the first 24 h, so the operation of PCA equipment can be difficult. We also decided to resign from additional NSAIDs prescription for patients in the postoperative period because of potential gastrointestinal and renal side effects.

Another limitation is that our study only compared lidocaine infusion with placebo but not with other types of analgesia, such as an epidural catheter. We do not use this type of analgesia in vascular surgery in our hospital, although it is widely adapted in ERAS protocols [19]. 

Although lidocaine is considered to be a safe drug, we did not assess its plasma level, which could prove indisputably the safety of the regimen in our study population. There are some single reports regarding the risk of side effects including postoperative delirium [30]. 

Finally, our study was underpowered to assess the complications of lidocaine infusion including POD—we only presented postoperative course data, which cannot be interpreted as conclusive. The small number of major medical events makes it impossible to perform the proper analysis.

## 6. Conclusions

In high-risk vascular surgery, perioperative lidocaine infusion as an adjunct to standard opioid-based analgesia is effective. It may decrease opioid consumption by more than 20% during the first 24 h after surgery, with no serious adverse effects noted during the study period.

## Figures and Tables

**Table 1:** Baseline patients’ characteristics. Continuous data were presented as the median and IQR or mean and 95%CI. Categorical data as total number and %.

	Lidocaine (n = 32)	Placebo (n = 35)	*p*-Value
**Age (years)**	64.4 (95%CI 62.4–66.4)	64.5 (95%CI 62.4–67)	0.91
**Male n (%)**	28/32 (88%)	28/35 (80%)	0.4
**Body weight (kg)**	78.6 (95%CI 73.5–83.7)	79.8 (95%CI 74.7–84.9)	0.74
**IBW (kg)**	69 (IQR 63.5–70.5)	68 (IQR 61–72)	0.56
**ASA-PS CLASS N (%)**			
**II**	9 (28%)	5 (14%)	0.23
**III**	23 (72%)	29 (83%)	0.28
**IV**	0	1 (3%)	0.99
**VQI CRI (%)**	3.25 (IQR 2.1–4.15)	3.6 (IQR 2.5–4.8)	0.29
**VQI RESP (%)**	8.25 (IQR 6.2–9.9)	6.2 (IQR 6.2–10.8)	0.96
**Nicotinism n (%)**	20/32 (62%)	20/35 (57%)	0.8
**Hypertension n (%)**	29/32 (91%)	28/35 (80%)	0.22
**CAD n (%)**	10/32 (31%)	18/35 (51%)	0.09
**Previous MI n (%)**	4/32 (12.5%)	4/35 (11%)	0.829
**T2DM n (%)**	2/32 (6%)	9/35 (26%)	0.03
**Previous IC n (%)**	1/32 (3%)	6/35 (17%)	0.1
**COPD n (%)**	12/32 (38%)	12/35 (34%)	0.78
**CKD 1–2 n (%)**	1/32 (3%)	4/35 (11%)	0.2

**IQR**—Inter-quartile range, 95%CI—95% Confidence Interval, **IBW**—Ideal body weight, **ASA-PS**—American Society Of Anesthesiologists Physical Status, **VQI CRI**—Vascular Quality Initiative Cardiac Risk Index, **VQI RESP**—Vascular Quality Initiative Respiratory Adverse Event Post Vascular Surgery, **CAD**—Coronary artery disease, MI—Myocardial infarction, T2DM—Type 2 diabetes mellitus **IC**—Stroke or transient ischemic attack, **COPD**—Chronic obstructive pulmonary disease, **CKD 1–2**—Chronic kidney disease stage 1–2.

**Table 2:** Characteristics of the intraoperative period and type of surgical procedures. Continuous data were presented as median and IQR. Categorical data as total number and %.

	Lidocaine (n = 32)	Placebo (n = 35)	*p*-Value
**Midazolam premedication**	3.75 (IQR 3.75–5.625)	3.75 (IQR 3.75–3.75)	0.68
**Intraoperative vital signs**			
Heart rate (/min)	65.5 (IQR 61.25–70.50)	66.0 (IQR 61.0–77.0)	0.64
Systolic blood pressure (mmHg)	121.5 (IQR 115.25–128.25)	121.5 (IQR 114.0–128.0)	0.95
Mean artery pressure (mmHg)	86.5 (IQR 81.75–90.25)	86.0 (IQR 79.0–90.0)	0.88
Response entropy (RE index)	36.0 (IQR 33.0–41.0)	38 (IQR 35.0–42.0)	0.23
State entropy (SE index)	35.5 (IQR 32.0–40.50)	37.0 (IQR 34.0–41.0)	0.29
**Types of surgical procedures**			
Abdominal aortic aneurysm resection n (%)	14/32 (44%)	22/35 (62.9%)	0.12
Aorto-femoral bypass n (%)	1/32 (3.1%)	1/35 (2.9%)	0.99
Aorto-biiliac bypass n (%)	6/32 (18.8%)	3/35 (8.6)	0.29
Aorto-bifemoral bypass n (%)	10/32 (31.3%)	7/35 (20%)	0.29
Ilio-femoral bypass n (%)	1/32 (3.1%)	2/35 (5.7%)	0.99
**Operating room times**			
Procedure time (min)	175 (IQR 150–202.5)	175 (IQR 155–215)	0.55
Aortic cross-clamping time (min)	59.5 (IQR 44–90)	56 (IQR 49–80)	0.91
General anesthesia time (min)	195.5 (IQR 174–231)	207 (IQR 176–254)	0.25

**Table 3:** Potential lidocaine side effects.

	Lidocaine (n = 32)	Placebo (n = 35)	*p*-Value
**Somnolence n (%)**	12/32 (37%)	16/35 (46%)	0.62
**Nausea and vomiting n (%)**	5/32 (16%)	4/35 (11%)	0.72
**Hypotension n (%)**	2/32 (6%)	7/35 (20%)	0.15
**Slurred speech n (%)**	2/32 (6%)	4/35 (11%)	0.67
**Dizziness n (%)**	1/32 (3%)	0/35 (0%)	0.96

**Table 4:** At 30-days follow up.

	Lidocaine (n = 32)	Placebo (n = 35)	*p*-Value
**Myocardial infarction n (%)**	0/32 (0%)	2/35 (5.7%)	0.49
**Cardiac arrest n (%)**	0/32 (0%)	0/35(0%)	-
**All-cause mortality n (%)**	0/32 (0%)	0/35 (0%)	-
**Mechanical ventilation n (%)**	1/32 (3.1%)	1/35 (2.9%)	0.99
**Pneumonia n (%)**	1/32 (3.1%)	2/35 (5.7%)	0.99
**Transfer to ICU n (%)**	1/32 (3.1%)	1/35 (2.9%)	0.99

## Data Availability

The data presented in this study are available on request from the corresponding author.
